# Treatment of Alcohols with Tosyl Chloride Does Not always Lead to the Formation of Tosylates

**DOI:** 10.3390/molecules16075665

**Published:** 2011-07-01

**Authors:** Rui Ding, Yong He, Xiao Wang, Jingli Xu, Yurong Chen, Man Feng, Chuanmin Qi

**Affiliations:** Key Laboratory of Radiopharmaceuticals, College of Chemistry, Beijing Normal University, Beijing 100875, China; Email: dingrui@mail.bnu.edu.cn (R.D.); heyong@mail.bnu.edu.cn (Y.H.); wangxiao@mail.bnu.edu.cn (X.W.); xujingli@mail.bnu.edu.cn (J.X.); chenyurong@mail.bnu.edu.cn (Y.C.); fengman@mail.bnu.edu.cn (M.F.)

**Keywords:** tosyl chloride, chlorination, tosylation, benzyl alcohols, pyridine methanols

## Abstract

Treatment of substituted benzyl alcohols with tosyl chloride resulted in the formation of the corresponding chlorides, not the usual tosylates. A series of experiments demonstrated that it was possible to predict whether chlorination or tosylation would occur for substituted benzyl alcohols and pyridine methanols. Treatment of electron withdrawing group-substituted benzyl alcohols with tosyl chloride gave the corresponding chlorides in moderate yields under mild conditions, which provided a simple way to directly prepare chlorides from alcohols.

## 1. Introduction

Alkyl tosylates are versatile substrates for substitution reactions. They contain an excellent leaving group and can be readily prepared from tosyl chloride and an alcohol, allow replacement of the hydroxy group by many good nucleophiles. Furthermore they are often crystalline solids that can be isolated and purified before further reaction. Tosylate is often a better leaving group than halide, as its preparation from an alcohol avoids stereochemical uncertainties and skeletal rearrangements. The tosylates can then be used in reactions with a variety of nucleophiles to give the corresponding nucleophilic substitution products [1]. Tosylates are commonly used as precusors for nucleophilic substitutions with [^l8^F]fluoride in radiopharmaceuticals. Tosyl chloride (TsCl), which is more reactive than tosyl anhydride and *p*-toluenesulfonyl acid, is the most widely used tosylating agent. Preparation of tosylates generally uses TsCl in the presence of a base, such as pyridine or triethylamine [2,3,4].

**Scheme 1:**
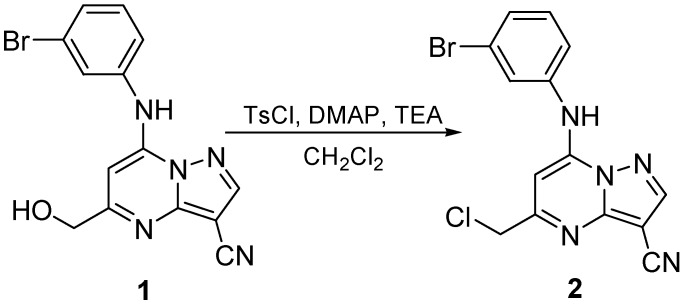
Formation of the chloride, not the desired tosylate.

Recently, as part of an intended ^l8^F radiolabeling synthetic route (depicted in the Supplementary Materials) we sought to prepare the corresponding tosylate by treatment of 5-hydroxy-7-(3-bromoanilino)-3-cyano-pyrazolo[1,5-a]pyrimidine (**1**) with TsCl (Scheme 1) under typical conditions [reaction of 1 mol equiv of an alcohol and 1.5 mol equiv of TsCl, with addition of a dichloromethane solution of catalytic amount of 4-dimethylaminopyridine (DMAP) and 1.5 mol equiv of triethylamine (TEA)]. Instead, we obtained the corresponding chloride **2**. Consequently we carried out a series of experiments and tried to discover why chlorination happened instead of tosylation. 

## 2. Results and Discussion

Treatment of *o*-, *m*- or *p*-nitro substituted benzyl alcohols with TsCl resulted in the formation of the corresponding chlorides in moderate yields (entry 1~3 in Table 1), among which *p*-nitrobenzyl alcohol gave in the highest yield (52%). 4-Nitrobenzyl bromide or iodide could be obtained by addition of sodium bromide (or sodium iodide) and 4-nitrobenzyl alcohol to the TsCl/DMAP/TEA mixture in dichloromethane (entry 4 and 5 in Table 1). The addition of sodium fluoride did not lead to the formation of 4-nitrobenzyl fluoride. The reactions of 4-bromobenzyl alcohol and 3-chlorobenzyl alcohol with TsCl also gave the corresponding chlorides in 30–35% yields (entry 6 and 7 in Table 1). In contrast, benzyl tosylate was produced in 53% yield when benzyl alcohol without any electron withdrawing substituent on the benzene ring reacted with TsCl (entry 8 in Table 1). The addition of KI to the reaction mixture of benzyl alcohol and TsCl led to the formation of benzyl iodide in 49% yield (entry 9 in Table 1).

The results could be rationalized by the following way. The reactions of benzyl alcohols with TsCl usually gave the corresponding tosylates initially. The nucleophilic substitution of tosyl group by Cl^−^ would not occur when no electron withdrawing inductive effect exists in tosylates, such as benzyl tosylate (entry 8 in Table 1) since Cl^−^ in the reaction mixture was a very fair nucleophile; but benzyl tosylate could be converted to benzyl iodide by a much better nucleophile I^−^ (entry 9 in Table 1). The reaction activity of benzyl tosylates could be improved when there was an electron withdrawing group, such as a nitro group, Br^−^ or Cl^−^, attached to the benzene ring due to the presence of electron withdrawing inductive effect. Thus the nucleophilic substitutions of these activated benzyl tosylates by Cl^−^ happened smoothly to give the corresponding chlorides despite the relatively poor nucleophilicity of Cl^−^ in the reaction mixture.

**Table 1:** Substituted benzyl alcohol chlorinations.

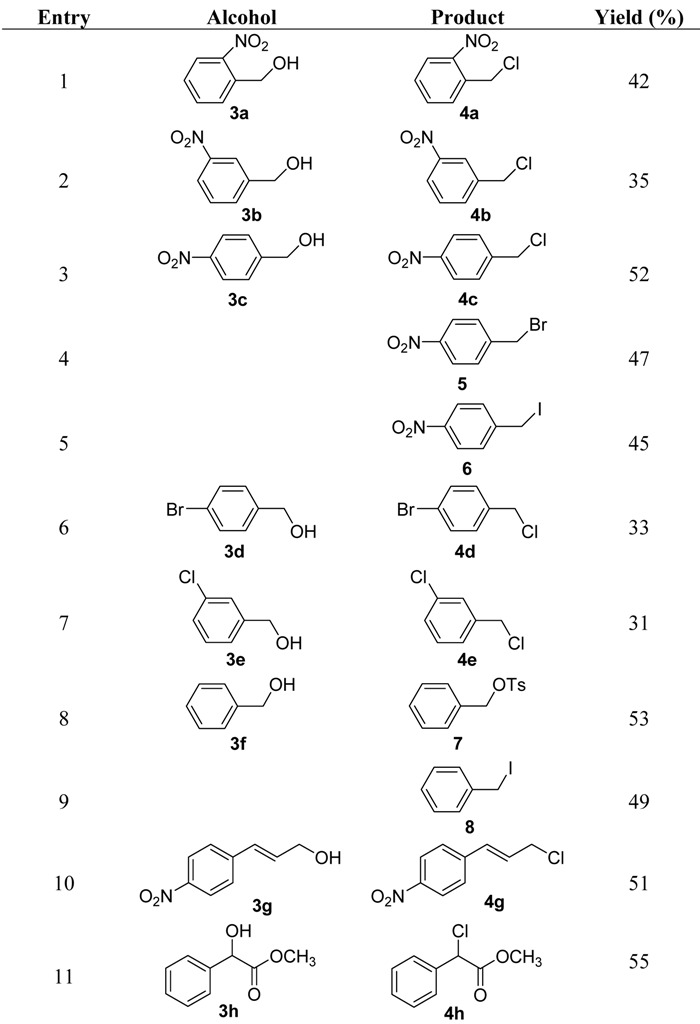

**Scheme 2:**
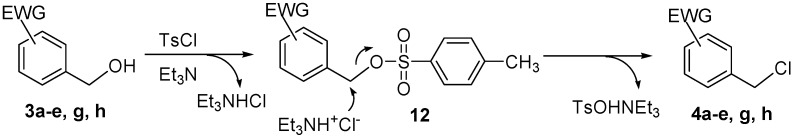
Possible reaction pathway.

The pathway of the formation of the benzyl chorides is depicted in Scheme 2. TsCl reacted initially with alcohol **3** to give tosylate **12**. TEA coordinated with the HCl byproduct to give triethylammonium hydrochloride and the nucleophilic chloride ion displaced the tosyl group to afford the substituted benzyl chloride **4**. The nitro substituted benzyl chlorides were usually produced in higher yields than bromo or chloro substituted benzyl chlorides, which might be due to the much stronger electron withdrawing effect of the nitro group. 4-Nitrocinnamyl alcohol reacted with TsCl to give 4-nitrocinnamyl chloride in 51% yield (entry 10 in Table 1), which could be explained by the same way as the formation of 4-nitrobenzyl chloride. Methyl mandelate was also used as a substrate and treated with TsCl to produce methyl 2-chloro-2-phenylacetate in 55% yield (entry 11 in Table 1), which indicated the intermediate tosylate was activated by the carbonyl group in the molecule and reacted with Cl^−^ to form the chloride.

Treatment of *p*-nitrobenzyl alcohol with *N,N*-dimethylformamide (DMF) instead of dichloromethane, resulted in the formation of *p*-nitrobenzyl chloride in 87% yield, according to [5]. The results could be explained by the effect that DMF as the polar aprotic solvent accelerated the nucleophilic substitution reaction.

Treatment of *p*-methylbenzyl alcohol with TsCl resulted in the formation of the corresponding chloride in 34% yield (entry 1 in Table 2). The reaction of *p*-methoxybenzyl alcohol with TsCl also gave the corresponding chloride in 38% yield (entry 2 in Table 2). 

**Table 2:** Substituted benzyl alcohol chlorinations.

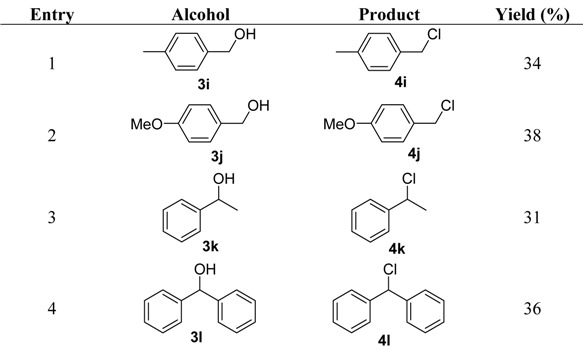

**Scheme 3:**
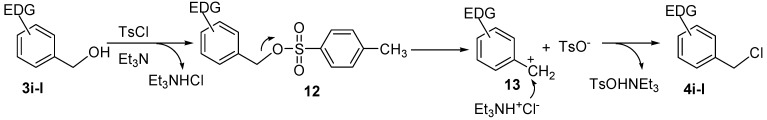
Possible reaction pathway.

Based on the above experiments, as depicted in Scheme 3, we envisaged that the formation of tosylate **12** *in situ* by treating the alcohol **3** with tosyl chloride in the presence of base, followed by loss of TsO^−^ generates a cation **13**, which was stabilized by the electron donating inductive effect of the methyl or methoxy group. The benzyl cation **13** was subjected to simultaneous nucleophilic attack by the chloride ion to afford the substituted benzyl chloride. The methoxybenzyl chloride was usually produced in higher yield than methylbenzyl chloride, which might be due to the stronger electron donating effect of methoxy group. 1-Phenylethanol reacted with TsCl to give 1-chloro-1-phenylethane in 31% yield (entry 3 in Table 2), which could be explained by the same way as the formation of *p*-methylbenzyl chloride. Diphenylmethanol was also used as a substrate to produce chlorodiphenylmethane in 36% yield (entry 4 in Table 2) upon treatment with TsCl, which indicated the benzyl cation was stabilized by the phenyl ring in the molecule and reacted with Cl^−^ to form the chloride. 

Treatment of 2-pyridinemethanol, 3-pyridinemethanol and 4-pyridinemethanol with TsCl resulted in the formation of the corresponding chlorides in relatively low yields (entry 1–3 in Table 3). The reaction of 4-nitro-2-pyridine methanol with TsCl also gave the corresponding chloride in 39% yield (entry 4 in Table 3). 

**Table 3:** Pyridine methanol chlorinations.

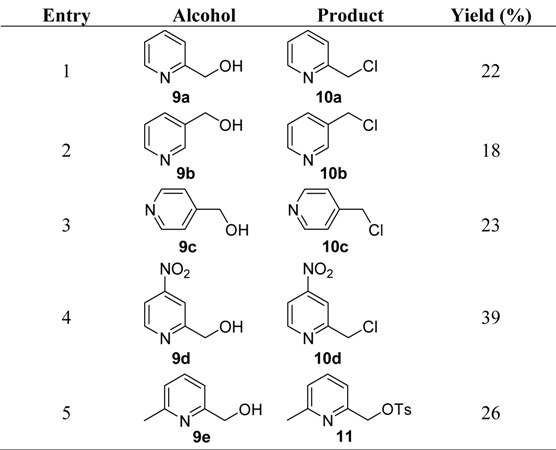

These results could be explained by the same way as benzyl alcohols. Pyridine methanols reacted with TsCl to give the corresponding tosylates at first. The tosylates were activated to some extent since the presence of the nitrogen atom in the pyridine ring distorted the electron distribution both in the π-bonding system and in the σ-bonds and a strong destabilizing inductive effect took over because the nitrogen atom was not directly attached to the electrophilic carbon. The nitro substituted pyridinemethyl chloride was usually produced in higher yield than pyridinemethyl chlorides, which might be due to the stronger electron withdrawing effect of nitro group. In contrast, treatment of 6-methyl-2-pyridinemethanol with TsCl led to the formation of 6-methyl-2-tosyloxy-methylpyridine (entry 5 in Table 3), which meant that the electron donating effect of methyl group offset the opposite effect of the nitrogen atom in the pyridine ring and the formed tosylate was not active enough to occur nucleophilic substitution by Cl^−^.

In this system, using TsCl as chlorination reagent was a particularly mild method for the transformation of alcohols to chlorides. In comparison with the traditional method for preparing chlorides by convertion of an alcohol to a mesylate followed by treatment with LiCl [6], using TsCl appeared to be a more convenient approach with respect to other reports [7,8,9].

## 3. Experimental

### 3.1. General

^1^H- and ^13^C-NMR spectra were recorded on a Bruker spectrometer operating in CDCl_3_ at 400 and 100 MHz, respectively. The IR spectra were recorded on a Nicolet-AVATAR 360 FT-IR spectrometer using KBr pellets in the 4000–400 cm^−1^ regions. ESI-MS were performed on Waters LCT Premier XE. EI-MS were performed on Bruker 320-MS.

### 3.2. General Procedure for Treatment of Alcohol with Tosyl Chloride

Alcohol (1 mmol), TEA (0.22 mL, 1.5 mmol) and DMAP (30 mg, 0.2 mmol) were added to CH_2_Cl_2_ (5 mL) at 0 °C. Then the solution of tosyl chloride (286 mg, 1.5 mmol) in CH_2_Cl_2_ (5 mL) was added dropwise. The reaction mixture was stirred at 0 °C for 30 min then at 15 °C for 12 h. Water (10 mL) was added, and then the organic phase was washed with saturated solution of NaHCO_3_ (10 mL × 2), followed by brine. The organic layer was collected and dried over Na_2_SO_4_, and the solvent evaporated under vacuum. The crude was purified by column chromatography (silica gel; petroleum ether/ethyl acetate = 3:1) to afford the desired compound.

*2-Nitrobenzyl chloride* (**4a**). ^1^H-NMR (CDCl_3_): δ 4.98 (2H, s), 7.51 (1H, m), 7.63–7.70 (2H, m), 8.05 (1H, d, *J* = 8.1 Hz). ^13^C-NMR (CDCl_3_): δ 42.7, 125.2, 129.4, 131.6, 132.4, 133.7, 148.0. MS (ESI): *m/z* = 171 [M^+^], 136 [M-Cl]^+^. 

*3-Nitrobenzyl chloride* (**4b**). ^1^H-NMR (CDCl_3_): δ 4.66 (2H, s), 7.56 (1H, t, *J* = 7.9 Hz), 7.73 (1H, d, *J* = 7.5 Hz), 8.18 (1H, dd, *J* = 7.0, 1.2 Hz), 8.27 (1H, s). ^13^C-NMR (CDCl_3_): δ 44.6, 123.3, 123.4, 129.8, 134.5, 139.4, 148.4. MS (ESI): *m/z* = 171 [M^+^], 136 [M-Cl]^+^. 

*4-Nitrobenzyl chloride* (**4c**). ^1^H-NMR (CDCl_3_): δ 4.65 (2H, s), 7.58 (2H, d, *J* = 8.7 Hz), 8.22 (2H, d, *J* = 8.7 Hz). ^13^C-NMR (CDCl_3_): δ 44.5, 123.9, 129.3, 144.3, 147.8. MS (ESI): *m/z* = 171 [M^+^], 136 [M-Cl]^+^, 125 [M-NO_2_]^+^. 

*4-Nitrobenzyl bromide* (**5**). ^1^H-NMR (CDCl_3_): δ 4.52 (2H, s), 7.57 (2H, d, *J* = 8.7 Hz), 8.20 (2H, d, *J* = 8.7 Hz). ^13^C-NMR (CDCl_3_): δ 30.9, 124.0, 129.9, 144.7, 147.8. MS (EI): *m/z* = 215 [M^+^], 136 [M-Br]^+^. 

*4-Nitrobenzyl iodide* (**6**). ^1^H-NMR (CDCl_3_): δ 4.48 (2H, s), 7.54 (2H, d, *J* = 8.7 Hz), 8.15 (2H, d, *J* = 8.7 Hz). ^13^C-NMR (CDCl_3_): δ 0.0, 122.1, 127.6, 144.8, 145.3. MS (EI): *m/z* = 263 [M^+^], 136 [M-I]^+^. 

*4-Bromobenzyl chloride* (**4d**). ^1^H-NMR (CDCl_3_): δ 4.53 (2H, s), 7.27 (2H, d, *J* = 8.4 Hz), 7.48 (2H, d, *J* = 8.4 Hz). ^13^C-NMR (CDCl_3_): δ 45.3, 122.5, 130.2, 131.9, 136.5. MS (EI): *m/z* = 204 [M^+^], 169 [M-Cl]^+^. 

*3-Chlorobenzyl chloride* (**4e**). ^1^H-NMR (CDCl_3_): δ 4.54 (2H, s), 7.25–7.32 (3H, m), 7.39 (1H, s). ^13^C-NMR (CDCl_3_): δ 45.2, 126.6, 128.5, 129.8, 130.3, 134.5, 139.3. MS (EI): *m/z* = 160 [M^+^], 125 [M-Cl]^+^. 

*Benzyl tosylate* (**7**). ^1^H-NMR (CDCl_3_): δ 2.44 (3H, s), 5.05 (2H, s), 7.24 (2H, m), 7.32 (5H, m), 7.78 (2H, d, *J* = 8.3 Hz). ^13^C-NMR (CDCl_3_): δ 21.6, 71.9, 127.1, 128.0, 128.5, 128.6, 129.0, 129.8, 133.3, 144.8. 

*Benzyl iodide* (**8**). ^1^H-NMR (CDCl_3_): δ 4.46 (2H, s), 7.21–7.31 (3H, m), 7.37 (2H, m). ^13^C-NMR (CDCl_3_): δ 5.6, 127.9, 128.7, 128.8, 139.3. MS (EI): *m/z* = 218 [M^+^], 91 [M-I]^+^. 

*4-Nitrocinnamyl chloride* (**4g**). ^1^H-NMR (CDCl_3_): δ 4.26 (2H, dd, *J* = 5.7, 1.1 Hz), 6.50 (1H, dt, *J* = 15.7, 6.8 Hz), 6.72 (1H, d, *J* = 15.7 Hz), 7.54 (2H, d, *J* = 8.8 Hz), 8.19 (2H, d, *J* = 8.8 Hz). ^13^C-NMR (CDCl_3_): δ 44.3, 124.0, 127.2, 129.6, 131.7, 142.3, 147.4. MS (EI): *m/z* = 197 [M^+^], 162 [M-Cl]^+^. 

*Methyl 2-chloro-2-phenylacetate* (**4h**). ^1^H-NMR (CDCl_3_): δ 3.77 (3H, s), 5.36 (1H, s), 7.36–7.41 (3H, m), 7.49 (2H, m). ^13^C-NMR (CDCl_3_): δ 53.3, 58.9, 127.9, 128.9, 129.3, 135.7, 168.8. MS (EI): *m/z* = 184 [M^+^], 125 [M-COOCH_3_]^+^. 

*4-Methylbenzyl chloride* (**4i**). ^1^H-NMR (CDCl_3_): δ 2.34 (3H, s), 4.55 (2H, s), 7.16 (2H, d, *J* = 8.0 Hz), 7.26 (2H, d, *J* = 8.0 Hz). ^13^C-NMR (CDCl_3_): δ 21.2, 46.3, 128.6, 129.4, 134.6, 138.3. MS (EI): *m/z* = 140 [M^+^], 105 [M-Cl]^+^.

*4-Methoxybenzyl chloride* (**4j**). ^1^H-NMR (CDCl_3_): δ 3.80 (3H, s), 4.56 (2H, s), 6.89 (2H, dm, *J* = 8.7 Hz), 7.29 (2H, dm, *J* = 8.7 Hz). ^13^C-NMR (CDCl_3_): δ 46.3, 55.3, 114.4, 129.7, 130.1, 159.7. MS (EI): *m/z* = 156 [M^+^], 121 [M-Cl]^+^.

*1-Chloro-1-phenylethane* (**4k**). ^1^H-NMR (CDCl_3_): δ 1.85 (3H, d, *J* = 6.8 Hz), 5.07 (1H, q, *J* = 6.8 Hz), 7.28 (1H, m), 7.34 (2H, m), 7.40 (2H, m). ^13^C-NMR (CDCl_3_): δ 26.5, 58.7, 126.5, 128.2, 128.6, 142.8. MS (EI): *m/z* = 140 [M^+^], 105 [M-Cl]^+^.

*Chlorodiphenylmethane* (**4l**). ^1^H-NMR (CDCl_3_): δ 5.40 (1H, s), 7.23–7.42 (10H, m). ^13^C-NMR (CDCl_3_): δ 80.0, 126.6, 127.3, 128.4, 142.2.

*2-(Chloromethyl)pyridine* (**10a**). ^1^H-NMR (CDCl_3_): δ 4.68 (2H, s), 7.25 (1H, m), 7.49 (1H, d, *J* = 7.8 Hz), 7.71–7.76 (1H, td, *J* = 7.7, 1.8 Hz), 8.58 (1H, dm, *J* = 4.2 Hz). ^13^C-NMR (CDCl_3_): δ 46.7, 122.9, 123.1, 137.2, 149.2, 156.6. MS (ESI): *m/z* = 128.1 [(M+H)^+^]. 

*3-(Chloromethyl)pyridine* (**10b**). ^1^H-NMR (CDCl_3_): δ 4.60 (2H, s), 7.33 (1H, dd, *J* = 3.0, 4.8 Hz), 7.74 (1H, dt, *J* = 7.9, 1.9 Hz), 8.58 (1H, dd, *J* = 3.3, 1.5 Hz), 8.63 (1H, d, *J* = 2.0 Hz). ^13^C-NMR (CDCl_3_): δ 43.1, 123.7, 133.3, 136.3, 149.4, 149.6. MS (ESI): *m/z* = 128.1 [(M+H)^+^]. 

*4-(Chloromethyl)pyridine* (**10c**). ^1^H-NMR (CDCl_3_): δ 4.55 (2H, s), 7.34 (2H, d, *J* = 5.9 Hz), 8.62 (2H, d, *J* = 5.9 Hz). ^13^C-NMR (CDCl_3_): δ 44.1, 123.0, 146.0, 150.1. MS (ESI): *m/z* = 128.1 [(M+H)^+^]. 

*2-(Chloromethyl)-4-nitro-pyridine* (**10d**). ^1^H-NMR (CDCl_3_): δ 4.81 (2H, s), 8.00 (1H, dd, *J* = 5.3, 2.0 Hz), 8.25 (1H, d, *J* = 1.9 Hz), 8.88 (1H, d, *J* = 5.3 Hz). ^13^C-NMR (CDCl_3_): δ 45.7, 115.4, 115.6, 151.6, 154.6, 160.0. MS (EI): *m/z* = 172 [M^+^]. 

*6-Methyl-2-tosyloxymethylpyridine* (**11**). ^1^H-NMR (CDCl_3_): δ 2.44 (3H, s), 2.48 (3H, s), 5.10 (2H, s), 7.08 (1H, d, *J* = 7.7 Hz), 7.23 (1H, d, *J* = 7.7 Hz), 7.34 (2H, d, *J* = 8.1 Hz), 7.57 (1H, t, *J* = 7.7 Hz), 7.82 (2H, d, *J* = 8.3 Hz). ^13^C-NMR (CDCl_3_): δ 21.5, 24.1, 71.9, 118.7, 122.8, 128.1, 129.8, 133.0, 137.1, 144.9, 153.1, 158.1. MS (ESI): *m/z* = 278.4 [(M+H)^+^]. 

## 4. Conclusions

In summary, we have shown that it was possible to predict whether chlorination or tosylation would occur for substituted benzyl alcohols and pyridine methanols. The procedure was very mild and operationally simple. For substituted benzyl alcohols, chlorination occurred when inductively electron withdrawing groups or inductively electron donating groups were attached to the benzene ring. For substituted pyridine methanols, tosylation occurred when inductively electron donating groups are attached to the pyridine ring. These conclusions were useful for designing organic compound synthesis, especially for ^l8^F labeled radiopharmaceuticals. Further exploration of the scope of TsCl chlorination or tosylation reactions is underway and will be reported in due course.

## Supplementary Materials

Supplementary File 1
