# Supplementary material for: Treatment of Alcohols with Tosyl Chloride Does Not always Lead to the Formation of Tosylates
Source: Molecules. 2011 Jul 1;16(7):5665–73. doi: 10.3390/molecules16075665 (PMC6264569; doi:10.3390/molecules16075665)
Supplement: Supplementary File 1 [file molecules-16-05665-s001.pdf]

Supplementary Materials

Scheme 4. Synthetic route of ^{18}F labeled precursor.

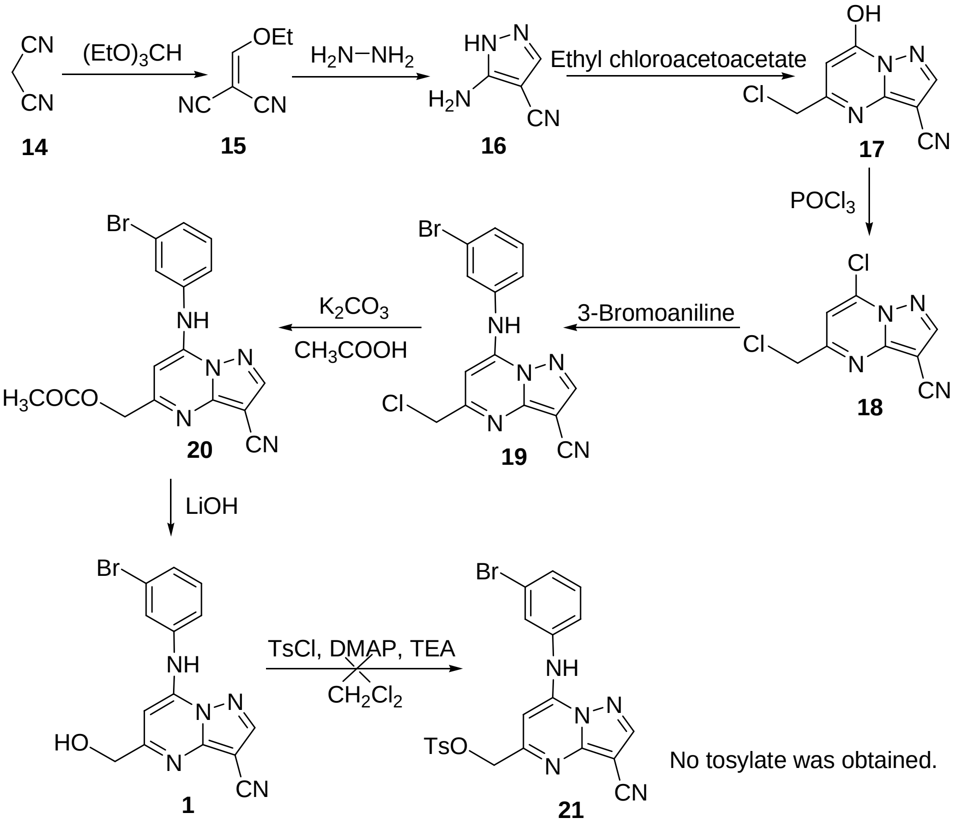

Synthesis of the ^{18}F labeled precursor **21** was performed according to the procedure outlined in Scheme 3.

2-(Ethoxymethylene)malononitrile **(15)**. Malononitrile (9.9 g, 0.150 mol), triethoxymethane (37.4 mL, 0.225 mol) and acetic anhydride (35.4 mL, 0.375 mol) were refluxed for 6 h. Then a little charcoal was added. The solvent was removed from the mixture by distillation. The hot residue was filtered and the filter cake was washed with hot ethanol, the filtrate was placed in a refrigerator overnight. After filtration, the crystalline was washed with ice-cold ethanol and dried to get the product **15** (17.4 g, 88%). m.p. 66–68 °C; IR (KBr, cm^{-1}): ν 3443, 3032, 2944, 2228, 1611, 1315, 1009, 883.

3-Amino-4-cyano-pyrazole **(16)**. Compound **15** (15 g, 0.123 mol) was carefully added to 85% hydrazine hydrate (12 mL, 0.248 mol) at room temperature. The resulting mixture was refluxed for 1 h. To the resulting solidified mass was added 10 mL of water. The solution stay in a refrigerator overnight and the mushy solution was filtered and the solid was washed with cold water and dried to get the product **16** (10.7 g, 81%). m.p. 174–176 °C; IR (KBr, cm^{-1}): ν 3416, 3342, 3147, 2955, 2238, 1645, 1570, 1522, 1221, 1034, 716.

5-Chloromethyl-3-cyano-7-hydroxypyrazolo[1,5-a]pyrimidine **(17)**. A mixture of **16** (9.0 g, 0.083 mol), ethyl chloroacetoacetate (24.51 g, 0.149 mol), and acetic acid (85 mL) was refluxed for 4 h. After cooling to room temperature, the pale white solid was filtered and washed with ethanol, dried with suction, and recrystallized from ethyl acetate to obtain the solid product **17** (15.93 g, 92%).

m.p. > 300 °C; IR (KBr, cm^{-1}): ν 3445, 3166, 3113, 3074, 2964, 2848, 2231, 1709, 1639, 1588, 1497, 1427, 1413, 1347, 1133, 748, 639.

5-Chloromethyl-3-cyano-7-chloropyrazolo[1,5-a]pyrimidine **(18)**. Phosphoryl trichloride (0.78 mL, 8.6 mmol) was added dropwise into a solution of **17** (1.19 g, 5.7 mmol) in anhydrous pyridine (0.5 g, 6.3 mmol). The mixture was heated to 85 °C slowly, and then stirred at 120 °C for 1 h. After it was cooled to 60 °C, chloroform (25 mL) was added and stirred for 1 h. Cold water (30 mL) was added to the solution. The insoluble materials was filtered off, the organic layer of the filtrate was washed with cold water to pH 7, concentrated, and recrystallized from ethyl acetate/cyclohexane to give the compound **18** (0.92 g, 71%). m.p. 119–120 °C; IR (KBr, cm^{-1}): ν 3440, 3129, 3091, 2233, 1613, 1542, 1499, 1456, 1406, 1369, 1346, 1290, 1269, 1220, 1183, 900, 825, 755, 744, 648.

5-Chloromethyl-3-cyano-7-(3-bromoanilino)-pyrazolo[1,5-a]pyrimidine **(19)**. A mixture of **18** (0.8 g, 3.5 mmol), 3-bromoaniline (0.8 g, 4.6 mmol), and isopropanol (10 mL) was heated at 60 °C for 2 h. After cooling to room temperature, a massive solid precipitated. Recrystallization from methol gave **19** as slight yellow powder (0.81 g, 66%). ^{1}H-NMR ($CDCl_3$, 400 MHz): δ 4.64 (2H, s), 6.74 (1H, s), 7.36 (1H, dm, *J* = 8.2 Hz), 7.42 (1H, t, *J* = 7.9 Hz), 7.52 (1H, dm, *J* = 8.0 Hz), 7.57 (1H, t, *J* = 1.9 Hz), 8.16 (1H, s), 8.33 (1H, s). ^{13}C-NMR ($CDCl_3$, 100 MHz): δ 46.1, 82.4, 88.3, 112.9, 122.5, 123.7, 127.1, 130.7, 131.5, 136.4, 145.7, 146.9, 149.9, 161.4. MS(ESI): m/z = 364.0 [$(M+H)^+$].

(7-(3-Bromoanilino)-3-cyanopyrazolo[1,5-a]pyrimidin-5-yl)methyl acetate **(20)**. An acetonitrile (5 mL) solution of potassium carbonate (345 mg, 2.5 mmol), tetrabutyl ammonium bromide (332 mg, 1 mmol) and acetic acid (120 mg, 2 mmol) was heated to reflux for 1.5 h. Then the compound **19** (362 mg, 1 mmol) was added and stirred for 1.5 h. The solvent was removed by rotary evaporation under vacuum. Dichloromethane was added and the organic phase was washed with saturated aqueous sodium bicarbonate, brine, dried over anhydrous sodium sulfate, filtered, and concentrated. The crude was purified by column chromatography (silica gel; petroleum ether/ethyl acetate = 4:1) to afford the desired compound **20** (320 mg, 83%). ^{1}H-NMR ($CDCl_3$, 400 MHz): δ 2.19 (3H, s), 5.20 (2H, s), 6.55 (1H, s), 7.33 (1H, dm, *J* = 7.8 Hz), 7.40 (1H, t, *J* = 8.0 Hz), 7.52 (1H, dm, *J* = 8.0 Hz), 7.58 (1H, t, *J* = 1.9 Hz), 8.14 (1H, s), 8.32 (1H, s). ^{13}C-NMR ($CDCl_3$, 100 MHz): δ 20.8, 65.9, 82.0, 86.9, 113.0, 122.4, 123.5, 126.9, 130.4, 131.4, 136.6, 145.4, 146.8, 150.2, 161.2, 170.3. MS(ESI): m/z = 388.1 [$(M+H)^+$].

5-Hydroxymethyl-3-cyano-7-(3-bromoanilino)-pyrazolo[1,5-a]pyrimidine **(1)**. LiOH (21 mg, 0.5 mmol) was added into the tetrahydrofuran (3 mL) solution of the compound **20** (77 mg, 0.2 mmol) and stirred at 0 °C for 30 min, then at room temperature for 120 min. The solvent was evaporated under vacuum, and dichloromethane was added. The organic phase was washed with dilute hydrochloric acid, saturated aqueous sodium bicarbonate, brine, dried over anhydrous sodium sulfate, filtered, and concentrated. Recrystallization from ethyl acetate and isopropanol gave **1** as slight yellow powder (65 mg, 95%). ^{1}H-NMR ($CDCl_3$, 400MHz): δ 3.36 (1H, t, *J* = 5.2 Hz), 4.76 (2H, d, *J* = 5.2 Hz), 6.47 (1H, s), 7.34 (1H, dm, *J* = 8.2 Hz), 7.40 (1H, t, *J* = 8.0 Hz), 7.52 (1H, dm, *J* = 8.0 Hz), 7.55 (1H, t, *J* = 1.9 Hz), 8.13 (1H, s), 8.31 (1H, s). ^{13}C-NMR ($CDCl_3$, 100 MHz): δ 62.7, 80.3, 84.4, 111.6, 121.2, 122.1, 125.7, 129.1, 129.9, 135.0, 143.8, 145.1, 148.5, 163.2. MS(ESI): m/z = 346.1 [$(M+H)^+$].
